# Case Report: Recurrent blepharal kaposiform hemangioendothelioma in an adult patient

**DOI:** 10.3389/fsurg.2023.1306566

**Published:** 2024-01-04

**Authors:** Wei Liu, Dan Zhao, Shirui Zhou, Hong Chen

**Affiliations:** Department of Ophthalmology, Union Hospital, Tongji Medical College, Huazhong University of Science and Technology, Wuhan, China

**Keywords:** kaposiform hemangioendothelioma, adult eyelid, recurrence, bleomycin, rare

## Abstract

**Background:**

Kaposiform hemangioendothelioma (KHE) is a rare and invasive vascular tumor that mainly occurs in children and is rarely seen in adults. We report a case of KHE found on the eyelid of an adult patient.

**Case report:**

We present an adult patient in whom KHE recurred 6 months after tumor resection. He underwent second surgical resection and intraoperative chemotherapy. There was no evidence of recurrence at the 3-year follow-up.

**Conclusion:**

KHE in adults is easy to be misdiagnosed. KHE can be treated by surgical resection. Complete resection of the tumor and intraoperative chemotherapy may help prevent a recurrence.

## 1 Introduction

Kaposiform hemangioendothelioma (KHE) is a rare vascular tumor commonly seen in infants, with a high incidence rate and mortality. The main pathological characteristics are abnormal angiogenesis and lymphangiogenesis (1). The vascular channels form a group of glomerular nodules with variable lumina and a surrounding pericytic layer (2). KHE often grows in an invasive way, showing dilated erythema or purple soft tissue mass, which is common in limbs and the trunk, followed by the retroperitoneum, and rarely in the head and neck (3, 4). There are not many treatment options for KHE; surgical resection is the main therapeutic option, but KHE often recurred after incomplete excision (5). Here we report a case of recurrent KHE in the eyelid of an adult male.

## 2 Case presentation

**Figure 1:**
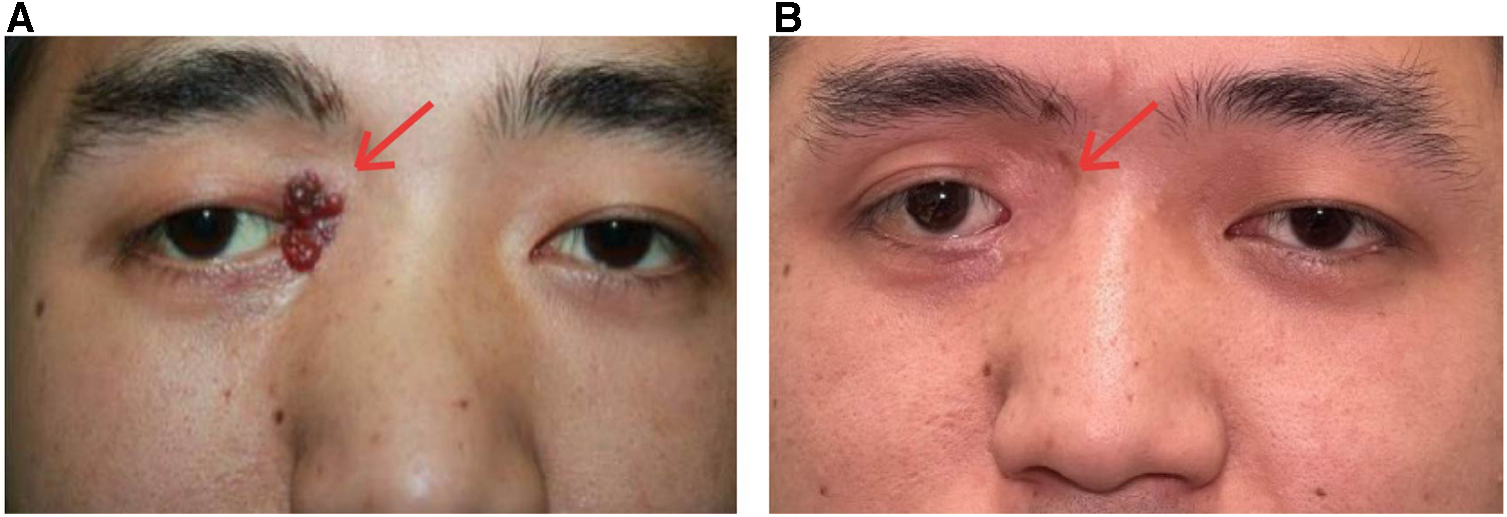
Surgical removal of the KHE combined with chemotherapy prevented the tumor recurrence in the 3-year follow-up. (**A**) Preoperation: the recurrent tumor had invaded the inner canthus and inner one-third part of the upper and lower eyelids. (**B**) The tumor was completely removed and combined with bleomycin treatment during the operation. No recurrence was noticed.

**Figure 2:**
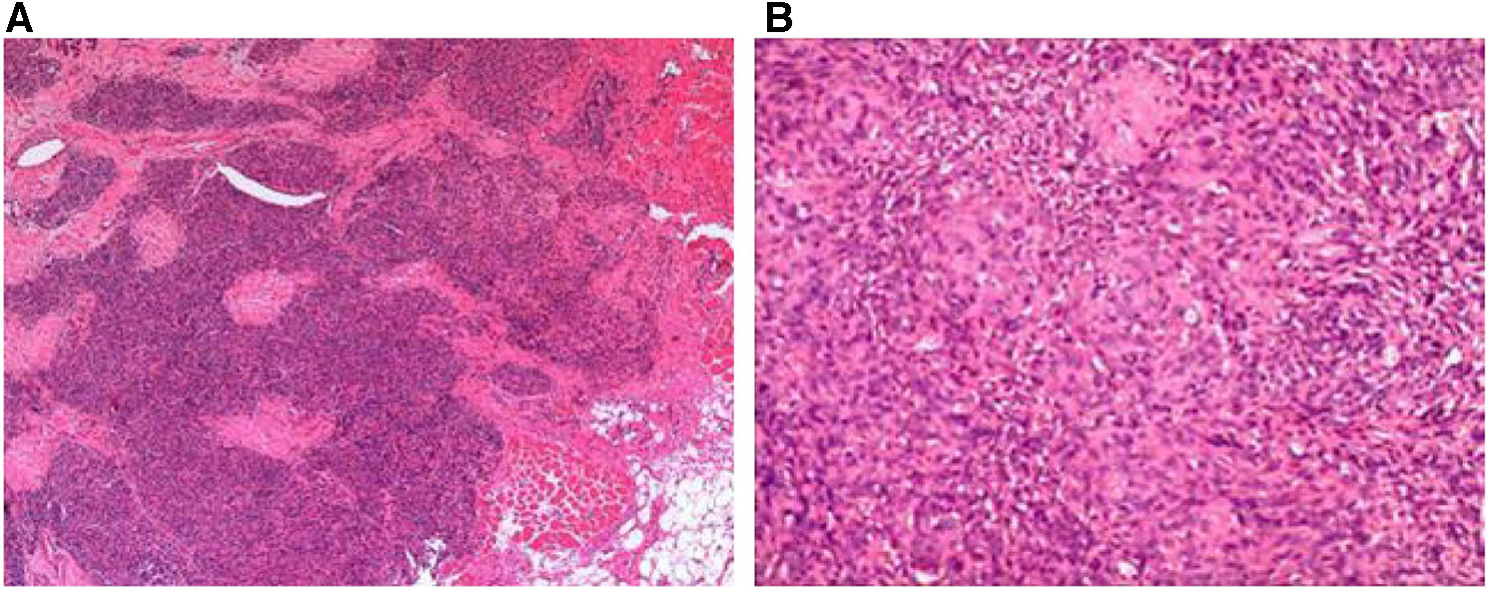
Histologic analysis of the mass. (**A**) Photomicrographs showing a nodular distribution of infiltrative angiomatous nodules [hematoxylin and eosin (H&E), original magnification: ×100]. (**B**) Angiomatous nodules were composed of crisscross spindle cell bundles and crescent vascular channels, with fibrin microthrombi in the lumen (H&E, original magnification: ×200).

A 23-year-old male patient with recurrent blepharal KHE was hospitalized 6 months after the first tumor resection. The tumor had an undefined reddish strawberry hyperplasia appearance, invading the inner canthus and inner one-third part of the upper and lower eyelids but did not cause the Kasabach–Merritt phenomenon (KMP) (Figure 1A). The patient was cured and was relapse-free after surgical resection of the tumor with accurate control of negative cutting edge and intraoperative prophylactic administration of bleomycin at the edge and the base of the tumor body. Histopathological findings confirmed a diagnosis of low-grade malignant kaposiform hemangioendothelioma (Figures 2A,B) with a positive stain of CD34 and negative HHV8. Blepharal kaposiform hemangioendothelioma in adults has been rarely reported. The effect of intraoperative application of anti-tumor agents such as bleomycin on the prevention of tumor recurrence remains to be further observed and studied.

No evidence of tumor recurrence was observed at the 3-year follow-up (Figure 1B).

## 3 Discussion

Standard therapy is yet to be established for recurrent KHE, because of its rarity and the associated difficulties in conducting clinical trials. Here we report such a rare case of KHE in the eyelid of an adult patient.

KHE is a rare invasive vascular tumor, which has no self-healing tendency and can cause red blood cell destruction, thrombocytopenia, and so on. KHE is classified as a vascular tumor with medium malignant potential by the World Health Organization (WHO) (2). KHE usually occurs in children's limbs and trunks, and is rarely observed in either adults or on the eyelid. About 42%–71% of KHE is known to be accompanied by KMP (1), which is a triad of microangiopathic hemolytic anemia, thrombocytopenia, and consumptive coagulation dysfunction (6, 7).

According to a 2016 study, only 26 cases of KHE in adults were found from 1993 to 2016, including only one case pertaining to the eyelid, and none of them was accompanied by KMP (8). The review by Schmid et al. confirmed that the incidence rate of KMP decreases with the age of patients (7). KMP is also not found in this case. However, even if there is no KMP, KHE should also be taken into account in the diagnosis when there is an enlarged purplish red mass, which is helpful in the early diagnosis and treatment to improve the prognosis of patients.

Clinical diagnosis of KHE is challenging due to the variety of clinical manifestations (3). The diagnosis requires an analysis of clinical presentations, laboratory examinations, and histopathological examinations (1). The gold standard for diagnosis is tissue biopsy; histologically, it is an isolated and fusional nodule formed by spindle-shaped endothelial cell bundles and crescent vascular channels, and the tumor margin is often ambiguous and irregular (9). In immunohistochemical staining, vascular endothelial markers of spindle cells, including CD31 and CD34, were positive (9). In this case, the diagnosis of KHE was made based on the combined pathological examination results from the first operation and the patient's clinical manifestations, Postoperative histopathological result confirmed the diagnosis of KHE.

Tufted angioma (TA), juvenile hemangioma (JH), infantile hemangioma (IH), and congenital hemangioma can be similar to KHE. Most of them can be differentiated by histology and immunophenotype (10). TA is a superficial benign tumor; some authors think that KHE and TA represent two ends of the histological spectrum, and now they have been combined (2). KHE is characterized by round nodules and red cell sequestration, but JH lacks these features (2). IH expresses the glucose transporter 1 (GLUT1), which is not found in KHE (10).

There is no systematic treatment for KHE. Surgical resection is the preferred treatment for KHE at present, but its application is limited due to the difficulty of total excision and incomplete resection may have a high risk of recurrence (10, 11). In addition to surgery, drug therapy can also be used. Corticosteroids and chemotherapy are first-line therapies, and recently, sirolimus has also been used for treatment (12, 13). In this case, the patient underwent his first tumor resection in another hospital, and the postoperative pathological examination results indicated KHE but no further therapy was administered. Recurrence was noted 6 months later, but the cause of recurrence was unknown. It has been reported that recurrence occurs in about 15% of patients even after surgical resection (11). Despite taking extra care to leave clear margins after each operation, there was a patient who experienced recurrences (14). During the second operation, we confirmed that the surgical margin was negative through frozen sections and added bleomycin for local chemotherapy. Considering that if KHE could not be completely resected through surgery, drug chemotherapy was generally recommended (12). There is a case showed that surgical resection combined with chemotherapy is effective for recurrent tumor, even in patients with stage IV cancer (15). Also, in the treatment of hemangiomas, intratumoral injection of bleomycin may have a sclerosing effect on potentially residual or recalcitrant tumor tissue (16). No recurrence was found in the follow-up after the operation.

## 4 Conclusion

In conclusion, we successfully treated an adult patient with recurrent blepharal KHE. In this case, surgical resection combined with chemotherapy was effective during the 3-year follow-up. It is important to select the appropriate treatment method for each individual case of a rare tumor. Further studies of treatments for recurrent blepharal KHE are warranted.

## Data Availability

The raw data supporting the conclusions of this article will be made available by the authors, without undue reservation.
